# Recurrence Patterns and Prognostic Determinants of Odontogenic Tumors Following Radical Surgical Management: A Retrospective Cohort Study

**DOI:** 10.7759/cureus.109358

**Published:** 2026-05-21

**Authors:** Mayur M Shingade, Pratyaksha Singh Panwar, Manish Kumar Munjal, Florence Priya, Priyanka Jadhav, Manish Kumar Chandraker

**Affiliations:** 1 Department of Oral and Maxillofacial Surgery, Jawahar Medical Foundation's Annasaheb Chudaman Patil Memorial Dental College, Dhule, IND; 2 Department of Dentistry, Government Doon Medical College, Dehradun, IND; 3 Department of Oral Pathology, Surendra Dental College and Research Institute, Sriganganagar, IND; 4 Department of Public Health Dentistry, Sri Sai College of Dental Surgery and Hospital, Hyderabad, IND; 5 Department of Prosthodontics, Bharati Vidyapeeth (Deemed to be University) Dental College and Hospital, Sangli, IND; 6 Department of Oral and Maxillofacial Surgery, Maitri College of Dentistry and Research Centre, Durg, IND

**Keywords:** odontogenic tumors, prognosis, recurrence, surgical margins, survival analysis

## Abstract

Introduction: Odontogenic tumors exhibit a wide spectrum of biological behaviors ranging from indolent benign lesions to aggressive malignant neoplasms with significant recurrence potential. Despite advances in surgical management, recurrence remains a major clinical challenge, particularly in tumors with aggressive histology and large sizes. Identifying reliable clinicopathological predictors is essential for optimizing treatment strategies and improving long-term outcomes. This study aimed to evaluate recurrence patterns and recurrence-free survival in odontogenic tumors and to identify independent predictors of recurrence following radical surgical treatment.

Materials and methods: This single-center retrospective cohort study was conducted using anonymized records of patients treated between January 2011 and December 2022. A total of 465 patients' records were screened, and 127 met the eligibility criteria. Data on demographic variables, tumor characteristics, treatment details, and outcomes were collected. The primary outcome was tumor recurrence, and recurrence-free survival was analyzed. Statistical analysis was performed using R software (The R Core Team, R Foundation for Statistical Computing, Vienna, Austria), employing Kaplan-Meier survival analysis, the log-rank test, and Cox proportional hazards and logistic regression models.

Results: Of 127 patients, 72 (56.6%) had benign tumors and 55 (43.4%) had malignant tumors. Recurrence occurred in 27 (21.26%) patients, with significantly higher rates in malignant tumors than in benign tumors (p < 0.001). The median time to recurrence was shorter for malignant tumors (18 months) than for benign tumors (32 months) (p < 0.001). Malignant tumor type (hazard ratio (HR) = 3.51), tumor size (HR = 1.29 per cm), lymph node metastasis (HR = 4.57), and positive surgical margins (HR = 9.62) were significant predictors (p < 0.001). Multivariable analysis confirmed that tumor type (odds ratio (OR) = 3.76), size (OR = 1.25), nodal status (OR = 4.41), and margin status (OR = 3.89) were independent predictors.

Conclusion: Recurrence in odontogenic tumors is primarily determined by tumor aggressiveness and the adequacy of surgical clearance. Malignant tumors, larger sizes, nodal involvement, and inadequate margins significantly increase the risk of recurrence. Careful preoperative evaluation and appropriate surgical planning are critical for improving long-term outcomes.

## Introduction

Odontogenic tumors constitute a diverse group of lesions arising from the tooth-forming apparatus and exhibit a wide spectrum of biological behaviors, ranging from indolent benign lesions to aggressive malignancies with metastatic potential [1]. Variations in proliferative activity among odontogenic tumors, as demonstrated by Ki-67 expression in ameloblastoma variants, reflect differences in biological aggressiveness and potential clinical behavior [2]. Recent updates in classification systems, particularly the World Health Organization (WHO) 2022 classification of head and neck tumors, have further refined the categorization of odontogenic neoplasms, emphasizing the need for evidence-based management strategies tailored to tumor biology [3].

Surgical resection remains the cornerstone of treatment for most odontogenic tumors, especially those demonstrating aggressive or infiltrative behavior. Radical surgical approaches, including segmental or marginal resections, are often advocated to achieve tumor-free margins and reduce the risk of recurrence [4]. However, recurrence continues to be a major concern, particularly in malignant odontogenic tumors and certain aggressive benign variants, such as solid/multicystic ameloblastoma [5]. The risk of recurrence is influenced by multiple clinicopathological factors, including tumor size, histological subtype, margin status, and presence of lymph node metastasis [6].

Understanding recurrence patterns and identifying reliable prognostic indicators are crucial for optimizing treatment planning, guiding surgical decision-making, and improving long-term patient outcomes. Comprehensive analyses integrating multiple variables in real-world clinical settings remain limited, particularly in the Indian population. Furthermore, the role of radical surgery in modifying recurrence-free survival across different tumor types requires further elucidation. Therefore, the present study aimed to evaluate recurrence patterns and recurrence-free survival in benign and malignant odontogenic tumors following radical surgical resection (resection with tumor-free margins) and to identify clinicopathological factors associated with recurrence and prognostic outcomes. The objectives were to compare the recurrence rates between benign and malignant tumors and to identify independent clinicopathological predictors of recurrence, including tumor type, size, lymph node status, and surgical margin status.

## Materials and methods

Study design and setting

This single-center retrospective cohort study was conducted in accordance with the Strengthening the Reporting of Observational Studies in Epidemiology (STROBE) guidelines for observational research [7]. This retrospective design enabled the evaluation of recurrence patterns and prognostic factors using previously recorded clinical data without influencing treatment protocols. The study was conducted in the Department of Oral and Maxillofacial Surgery at Jawahar Medical Foundation’s Annasaheb Chudaman Patil Memorial Dental College, Dhule, India. The clinical records of patients treated between January 2011 and December 2022 were retrieved and analyzed. Data extraction and statistical analyses were performed between January 2025 and June 2025. A minimum follow-up period of 24 months was ensured for all included patient records or until the occurrence of documented recurrence.

This study utilized retrospective anonymized patient records, and no identifiable patient information was accessed at any stage. The requirement for informed consent was waived because there was no need for direct patient involvement. Furthermore, due to the use of fully de-identified archival data and the non-interventional nature of the study, ethical approval was waived by the institutional authority in accordance with accepted ethical standards for retrospective record-based studies. Strict confidentiality and data protection protocols were maintained throughout the study period.

Record selection criteria

Eligibility criteria were predefined. Inclusion criteria comprised patients with a histopathologically confirmed diagnosis of a primary odontogenic tumor, classified according to the WHO classification of head and neck tumors, who underwent radical surgical treatment [3]. Radical surgery was defined as resection with tumor-free margins, including marginal or segmental resection; for benign tumors, a minimum of 1 cm of uninvolved bone beyond the lesion; and for malignant tumors, at least 1.5 cm margins along with appropriate neck dissection when indicated [6]. Patients were required to have complete clinicopathological and radiographic records and a minimum follow-up period of 24 months or documented recurrence.

Radical surgical procedures were categorized as marginal or segmental resections based on the extent of bone involvement. Marginal resection involved the removal of the tumor with a surrounding margin of uninvolved bone while preserving mandibular continuity. In contrast, segmental resection involved en bloc removal of the tumor along with a full-thickness segment of the jaw, resulting in discontinuity defects, and was performed in cases with extensive cortical perforation, soft tissue invasion, or aggressive malignant behavior. The choice of surgical technique was determined based on tumor size, location, radiographic extent, and intraoperative assessment of tumor infiltration. All procedures were performed by experienced oral and maxillofacial surgeons following standard oncological principles.

Exclusion criteria included patients previously treated at another institution, syndromic cases (such as Gorlin-Goltz syndrome and Gardner syndrome), recurrent tumors at presentation, non-neoplastic odontogenic lesions, unicystic or peripheral ameloblastoma, and cases with incomplete data on key variables (tumor size, histological subtype, and margin status) or inadequate follow-up.

A census-based sampling approach was employed, which included all eligible cases available within the institutional database during the study period. A total of 465 cases of odontogenic tumors treated with radical surgery were initially screened, of which 127 cases fulfilled the eligibility criteria and were included in the final analysis. As the study encompassed the entire accessible population, formal sample size calculation was not required.

Data were extracted from electronic medical records and institutional pathology databases in a standardized format. The variables recorded included demographic characteristics (age and sex), tumor-related factors (type, size, location, and histological subtype), lymph node status, surgical margin status, and follow-up outcomes. The tumor size was measured radiographically using preoperative imaging. The margin status and histopathological diagnosis were obtained from the final pathology reports.

To minimize potential discrepancies in histopathological classification over the 11-year study period, all available archival histopathological slides and pathology records were retrospectively reviewed and reclassified by an experienced oral and maxillofacial pathologist. This re-evaluation was undertaken to ensure diagnostic standardization and consistency across the study cohort. Classification of benign and malignant odontogenic tumors, including aggressive variants such as solid/multicystic ameloblastoma, was therefore based on contemporary histopathological criteria to reduce classification bias and improve the biological consistency of recurrence and survival analyses.

Outcome variables

The primary outcome was tumor recurrence, which was defined as the reappearance of the tumor at the primary site, regional lymph nodes, or distant sites. The primary time-to-event endpoint was recurrence-free survival, calculated from the date of surgery to the first documented recurrence or last follow-up (censored cases).

Statistical analysis

Statistical analysis was performed using the R statistical software (version 4.3.2; R Foundation for Statistical Computing, Vienna, Austria). Continuous variables are expressed as mean ± standard deviation or median with interquartile range (IQR), while categorical variables are presented as frequencies and percentages. Group comparisons were performed using the Student’s t-test or Mann-Whitney U test for continuous variables and the chi-square test or Fisher’s exact test for categorical variables, as appropriate. Recurrence-free survival was estimated using the Kaplan-Meier method and compared using the log-rank test. Univariate and multivariate Cox proportional hazards regression analyses were performed to identify the predictors of recurrence, including tumor type, tumor size, lymph node status, margin status, age, sex, and tumor location. The results were expressed as hazard ratios (HRs) with 95% confidence intervals (CIs). Additionally, a multivariable binary logistic regression model was constructed to determine the independent predictors of recurrence. Statistical significance was set at p < 0.05.

## Results

**Figure 1:**
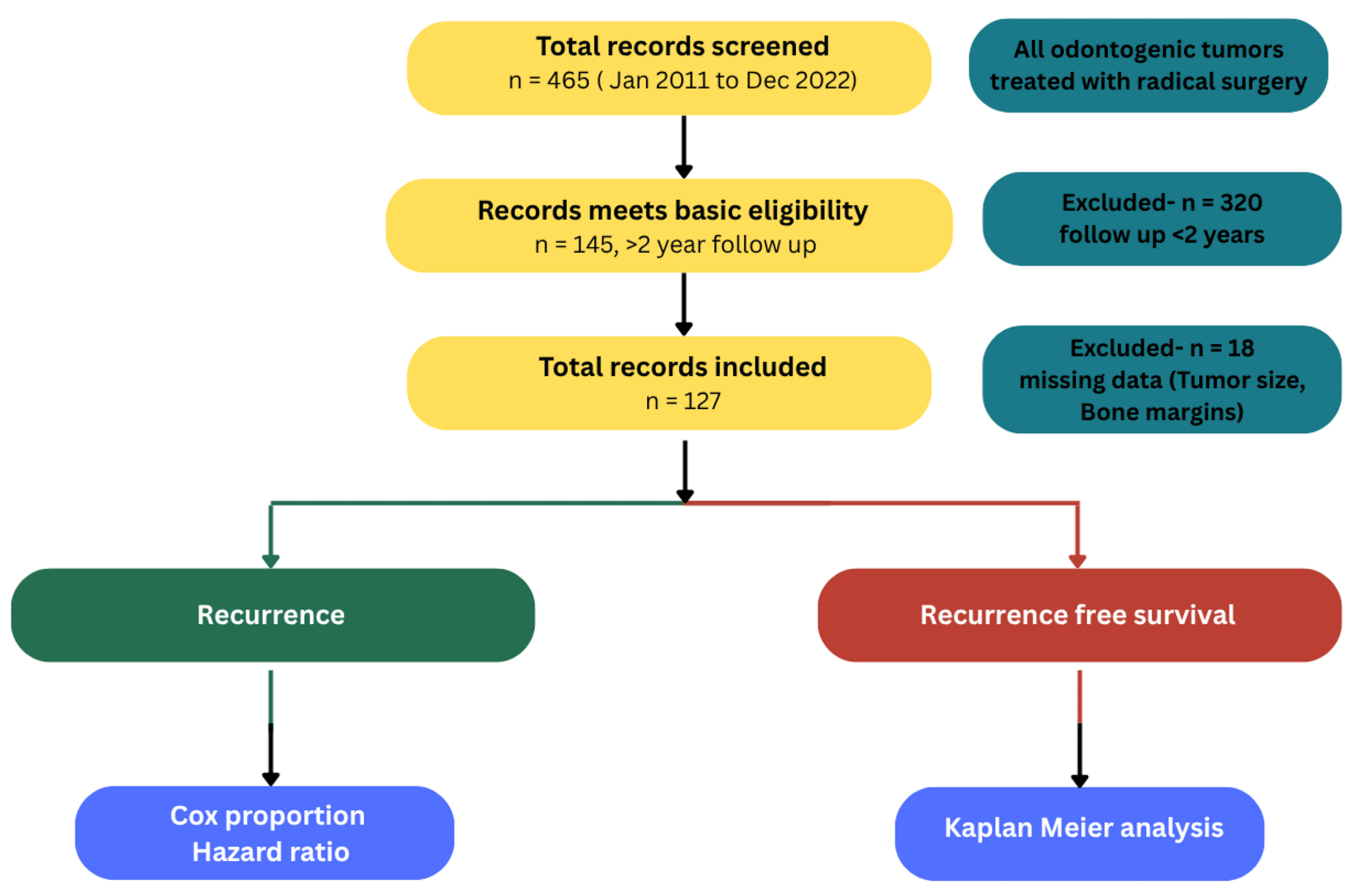
Screening of the included cases.

A total of 465 odontogenic tumor cases were screened, of which 127 (27.3%) met the eligibility criteria and were included in the final analysis (Figure 1).

Among these, 72 (56.6%) were benign, and 55 (43.4%) were malignant odontogenic tumors. As summarized in Table 1, patients with malignant tumors were significantly older than those with benign tumors (mean age: 50.2 ± 19.8; p = 0.018) and presented with significantly larger lesions (median: 5.9 cm; p = 0.032). Lymph node metastasis was exclusively observed in malignant tumors (p < 0.001). Histological subtype distribution differed significantly between the groups (p < 0.001), with solid/multicystic ameloblastoma predominating in benign lesions, whereas ameloblastic carcinoma and keratocystic odontogenic tumors were more frequent in the malignant group. No significant differences were noted in sex or tumor location. The median follow-up duration was significantly longer in patients with benign tumors (52 vs. 42 months; p = 0.041).

**Table 1:** Baseline clinicopathological characteristics of the study cohort. Values are presented as mean ± standard deviation (SD), median with interquartile range (IQR), or frequency with percentage (n%), Statistical tests used: Student’s t-test for continuous parametric data, Mann–Whitney U test for non-parametric data, and chi-square (χ²) test or Fisher’s exact test for categorical variables; *p < 0.05 considered statistically significant.

Characteristic	Benign odontogenic tumors (n = 72)	Malignant odontogenic tumors (n = 55)	Statistical test	p-value
Age (years, mean ± SD)	42.4 ± 16.5	50.2 ± 19.8	t = 3.27	0.018*
Sex, n (%)				
Male	38 (52.8)	30 (54.5)	χ^2^ = 0.04	0.84
Female	34 (47.2)	24 (43.6)		
Tumor location, n (%)				
Mandible	58 (80.6)	40 (72.7)	χ^2^ = 1.08	0.267
Maxilla	14 (19.4)	15 (27.3)		
Tumor size (cm, median (IQR))	5.2 [3.9–6.8]	5.9 [4.8–7.5]	U = 1540	0.032*
Histological subtype, n (%)				
Solid/multicystic ameloblastoma	62 (86.1)	26 (47.3)	χ^2^ = 24.76	< 0.001*
Keratocystic odontogenic tumors	6 (8.3)	16 (29.1)		
Ameloblastic carcinoma	2 (2.8)	12 (21.8)		
Lymph node metastasis, n (%)	0 (0.0)	9 (16.4)	Fisher's exact	< 0.001*
Follow-up (months, median (IQR))	52.0 (36.0–84.0)	42.0 (30.0–60.0)	U=1304	0.041*

The recurrence outcomes are detailed in Table 2. Overall, recurrence occurred in 27 (21.3%) cases, with a significantly higher rate in malignant tumors than in benign tumors (p < 0.001). Local recurrence was the most common pattern in both groups, with no significant differences between the groups. However, regional recurrence was observed only in malignant tumors (p = 0.010). Distant metastasis was rare and comparable between the groups. The median time to recurrence was significantly shorter for malignant tumors (18 months) than for benign tumors (32 months; p < 0.001).

**Table 2:** Comparison of recurrence patterns between benign and malignant odontogenic tumors. Values are presented as frequency with percentage (n%) or median with interquartile range (IQR), Percentages for recurrence patterns were calculated among recurrence cases only, Statistical tests used: chi-square (χ²) test, Fisher’s exact test (for small sample sizes), and Mann–Whitney U test, *p < 0.05 considered statistically significant.

Parameter		Benign odontogenic tumors (n = 72)	Malignant odontogenic tumors (n = 55)	Statistical test	p-value
Recurrence events, n (%)		8 (11.1)	19 (34.5)	χ² = 10.23	< 0.001*
Mode of recurrence, n (%)	Local recurrence only	7 (87.5)	12 (63.2)	Fisher's exact	0.240
	Regional recurrence	0 (0.0)	5 (26.3)	Fisher's exact	0.010*
	Distant metastasis	1 (12.5)	2 (10.5)	Fisher's exact	0.640
Time to recurrence (months, median (IQR))		32.0 (25.0–48.0)	18.0 (14.0–24.0)	U ≈ 1559	< 0.001*

**Figure 2:**
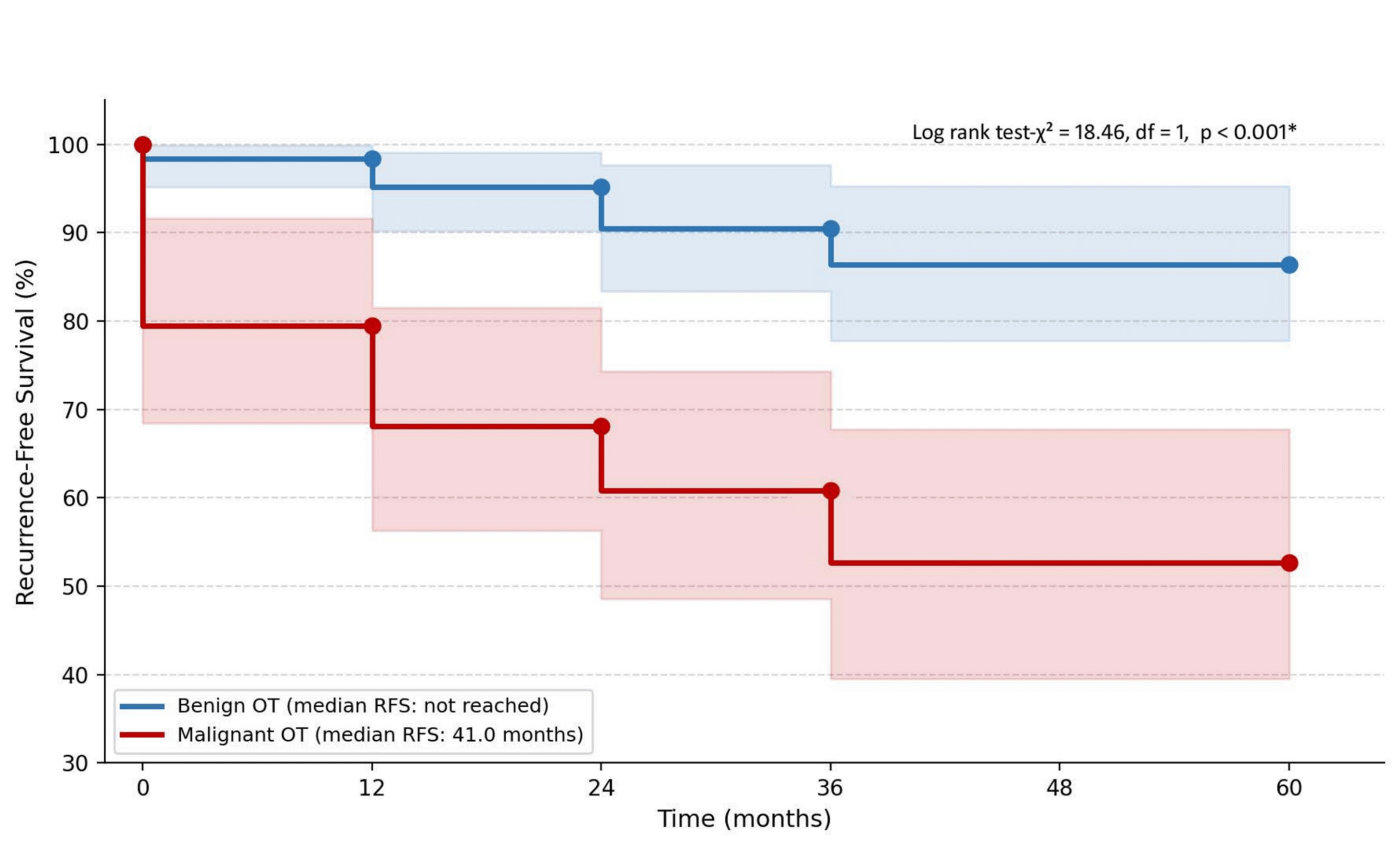
Kaplan–Meier curves for RFS in benign and malignant OTs. Df: degree of freedom, OT: odontogenic tumor, RFS: recurrence-free survival, time calculated from date of surgery to first recurrence or last follow-up, differences between groups compared using the log-rank test. The figure was generated using R statistical software (version 4.3.2; R Foundation for Statistical Computing, Vienna, Austria).

Kaplan-Meier survival analysis (Figure 2) demonstrated significantly better recurrence-free survival in benign tumors than in malignant tumors. The benign group maintained a recurrence-free survival of approximately 86% at 60 months, whereas the malignant group showed a progressive decline, with a median recurrence-free survival of 41 months.

The univariate Cox regression analysis (Table 3) identified several significant predictors of recurrence. The malignant tumor type was associated with a 3.5-fold increased risk of recurrence (HR = 3.51, p < 0.001). Tumor size was also a significant factor, with each 1 cm increase increasing recurrence risk by 29% (HR = 1.29, p < 0.001). Tumors > 6 cm were at the highest risk (HR = 3.86). Lymph node metastasis (HR = 4.57, p < 0.001) and positive surgical margins (HR = 9.62, p < 0.001) were observed. Age, sex, and tumor location were not statistically significant.

**Table 3:** Univariate Cox proportional hazards analysis for predictors of recurrence. HR: hazard ratio; CI: confidence interval, reference category shown for comparison, Cox proportional hazards model used, *p < 0.05 considered statistically significant, dashed lines (—) indicate reference categories or continuous parameters (events cannot be calculated).

Predictor variable		Events / Total N (%)	HR (Unadjusted)	95% CI	p-value
Tumor type	(malignant vs. benign)	19/55 (34.5) vs. 8/72 (11.1)	3.51	1.98–6.83	< 0.001*
Tumor size	(per 1 cm increase)	—	1.29	1.11–1.48	< 0.001*
Tumor size category	≤ 4.0 cm	5/51 (9.8)	Reference	—	—
	4.1–6.0 cm	12/46 (26.1)	2.45	1.45–4.65	0.008*
	>6.0 cm	10/30 (33.3)	3.86	2.09–7.98	< 0.001*
Lymph node metastasis	(pN+ vs. pN0)	6/9 (66.7) vs. 21/118 (17.8)	4.57	2.98–8.95	< 0.001*
Bony margin status	Negative (≥5 mm)	8/88 (9.1)	Reference	—	—
	Close (<5 mm negative)	8/22 (36.7)	3.61	2.02–6.91	< 0.001*
	Positive	11/14 (78.6)	9.62	5.49–15.32	< 0.001*
Age at diagnosis	(per 10-yr increase)	—	1.12	0.94–1.33	0.210
Sex	(male vs. female)	18/68 (26.5) vs. 9/59 (15.3)	1.55	0.93–2.59	0.090
Tumor location	(maxilla vs. mandible)	8/19 (42.1) vs. 19/108 (17.6)	2.17	1.54–4.46	0.103

Multivariable logistic regression (Table 4) confirmed that tumor type, tumor size, lymph node metastasis, and margin status were independent predictors of recurrence. Malignant tumors had nearly four-fold higher odds of recurrence (OR = 3.76, p < 0.001). Each 1 cm increase in tumor size increased the recurrence odds by 25% (p = 0.018). Lymph node metastasis (OR = 4.41) and close/positive margins (OR = 3.89) were also significant predictors (both p < 0.001).

**Table 4:** Multivariable logistic regression model for predictors of recurrence. OR: odds ratio; CI: confidence interval, all variables entered simultaneously in multivariable model, model calibration assessed using Hosmer–Lemeshow test (p = 0.62), Nagelkerke R² = 0.44, *p < 0.05 considered statistically significant; pN+: pathologically positive lymph nodes; pN0: absence of lymph node metastasis.

Predictor variable	OR (Adjusted)	95% CI	p-value	Interpretation
Tumor type (malignant vs. benign)	3.76	1.81–7.60	< 0.001*	3.8× higher odds of recurrence
Tumor size (per 1 cm increase)	1.25	1.04–1.51	0.018*	25% increase per cm
Lymph node metastasis (pN+ vs. pN0)	4.41	2.01–9.40	< 0.001*	4.4× higher odds
Bony margin status (close/positive vs. negative)	3.89	1.90–7.85	< 0.001*	3.9× higher odds
Model fit: Hosmer–Lemeshow χ² = 6.24, p = 0.62 (acceptable fit); Nagelkerke R² = 0.44				

**Figure 3:**
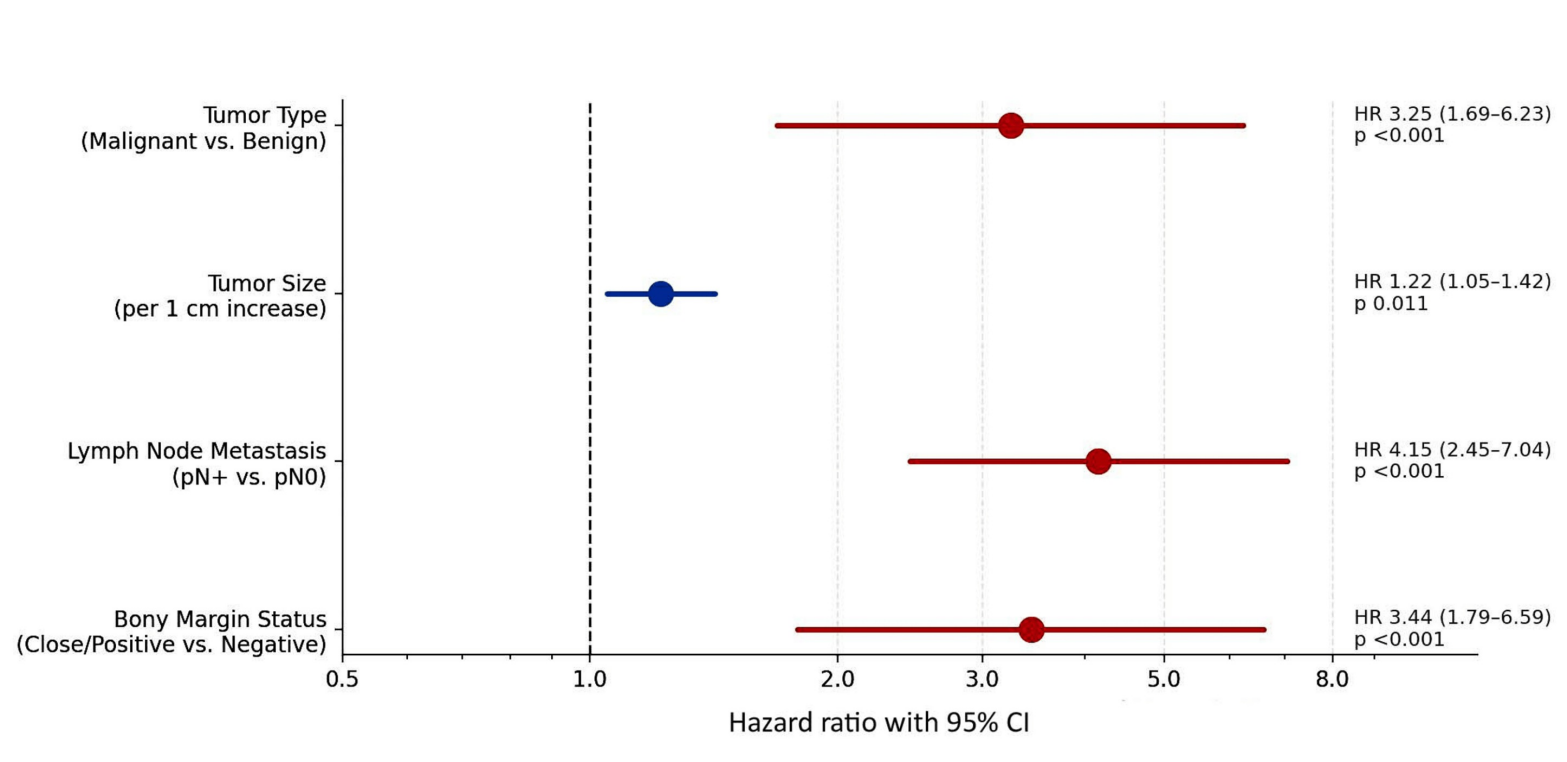
Multivariable Cox proportional hazards model for predictors of recurrence-free survival. HR: hazard ratio; CI: confidence interval Reference categories include benign tumor type, negative margin status, and absence of lymph node metastasis (pN0); pN+ means pathologically positive lymph nodes, values greater than 1 indicate increased risk of recurrence, error bars represent 95% CIs, and statistical significance was considered at p < 0.05. The figure was generated using R statistical software (version 4.3.2; R Foundation for Statistical Computing, Vienna, Austria).

The multivariable Cox proportional hazards model (Figure 3) further demonstrated that all four variables significantly reduced recurrence-free survival, with lymph node metastasis emerging as the strongest predictor, followed by margin status, malignant tumor type, and tumor size. Overall, these findings indicate that malignant tumor biology, larger tumor size, nodal involvement, and inadequate surgical margins are the key determinants of recurrence and survival outcomes in odontogenic tumors.

## Discussion

This retrospective cohort study evaluated recurrence patterns and prognostic determinants in odontogenic tumors following radical surgical management, providing clinically relevant insights into factors influencing recurrence and survival outcomes. The findings demonstrated that malignant tumor type, larger tumor size, lymph node metastasis, and compromised surgical margins are significant predictors of recurrence, highlighting the multifactorial nature of tumor behavior.

A key observation of this study was the significantly higher recurrence rate of malignant odontogenic tumors than of benign lesions. This finding is consistent with the known biological aggressiveness of malignant odontogenic neoplasms, which exhibit infiltrative growth patterns, higher proliferative indices, and a greater propensity for regional and distant spread [8]. Previous studies have emphasized that malignant variants such as ameloblastic carcinoma demonstrate increased mitotic activity and invasive potential, contributing to poorer outcomes [9]. These biological characteristics explain the earlier and more frequent recurrence observed in the malignant group in the present study, as reflected by the significantly shorter recurrence-free survival.

Tumor size has emerged as an important determinant of recurrence. Larger tumors were associated with increased recurrence risk in both univariate and multivariate analyses. This can be attributed to the likelihood of microscopic tumor extensions beyond radiographically visible margins, which makes complete surgical clearance challenging. Similar findings have been reported in previous studies, where tumor sizes greater than 4-6 cm were associated with increased recurrence rates and the need for more aggressive surgical approaches [10,11]. Larger lesions are also more likely to involve adjacent anatomical structures, thereby increasing the surgical complexity and risk of residual disease.

In the present study, lymph node metastasis was identified as the strongest independent predictor of recurrence. The presence of nodal involvement reflects advanced disease and a higher tumor burden, often indicating aggressive tumor biology [12]. This finding aligns with oncological principles, in which nodal metastasis is a well-established adverse prognostic factor in head and neck malignancies [13]. The exclusive occurrence of regional recurrence in malignant tumors further supports the role of lymphatic spread in disease progression and highlights the importance of appropriate neck evaluation and management in such cases.

Surgical margin status also had a significant impact on recurrence outcomes. Patients with close or positive margins had markedly higher recurrence rates, emphasizing the critical importance of achieving adequate tumor-free margins during resection. This observation is consistent with previous studies that reported significantly lower recurrence rates following radical surgery with clear margins compared to conservative approaches [6,14]. Incomplete excision allows residual tumor cells to persist, leading to local recurrence, which is the most common pattern observed in both benign and malignant tumors.

Interestingly, demographic factors, such as age and sex, were not significantly associated with recurrence in the present study. Although malignant tumors were more frequently observed in older patients, age did not independently influence recurrence risk, suggesting that intrinsic tumor biology, rather than patient-related factors, plays a more decisive role in determining clinical outcomes. Similar observations have been reported by Abdul-Aziz et al. [15], who demonstrated that malignant transformation and recurrence in odontogenic tumors are more closely linked to biological behavior than to demographic variables. Furthermore, the tumor location (mandible vs. maxilla) did not reach statistical significance in our analysis. While some case reports suggest that maxillary tumors may behave more aggressively owing to anatomical proximity to vital structures and delayed detection, this association was not evident in the present cohort, indicating that the site alone may not be an independent predictor when other pathological factors are accounted for [16,17].

Kaplan-Meier survival analysis further reinforced these findings, demonstrating significantly better recurrence-free survival in benign tumors than in malignant ones. The gradual decline in survival probability in the malignant group reflects the ongoing risk of recurrence over time, underscoring the need for long-term surveillance of these patients. The multivariate Cox model confirmed that all key variables independently influenced recurrence-free survival, validating their prognostic significance.

From a clinical perspective, the results of this study have important implications in treatment planning. The identification of high-risk features, such as large tumor size, nodal metastasis, and close or positive margins, supports the use of more aggressive surgical strategies in selected cases to achieve optimal disease control. Radical resection with adequate margins remains the cornerstone of management, particularly for malignant tumors and aggressive benign variants [14,18]. At the same time, careful preoperative assessment using advanced imaging and histopathological evaluation is essential to guide surgical decision-making. These findings also emphasize the importance of individualized treatment planning that balances oncological safety with functional and aesthetic outcomes.

This study has several limitations. The retrospective single-center design may introduce selection bias and variability in data completeness. Histopathological classification was based on available archival records and may have been influenced by evolving WHO classification criteria over time. Information regarding postoperative adjuvant therapy, including radiotherapy and chemotherapy, was not consistently available and therefore could not be incorporated into the recurrence analysis. Competing-risk analysis was not performed because uniformly documented cause-specific mortality data were unavailable. In addition, formal assessment of the proportional hazards assumption and internal validation of the Cox regression models were not undertaken, as the survival analyses were primarily exploratory in nature. Variability in follow-up duration and incomplete documentation regarding loss to follow-up may also have influenced recurrence estimates. Therefore, the findings should be interpreted with appropriate caution.

## Conclusions

This study demonstrated that recurrence of odontogenic tumors was strongly influenced by tumor biology and surgical factors rather than demographic variables. Malignant tumor type, larger tumor size, lymph node metastasis, and close or positive surgical margins were identified as independent predictors of recurrence and reduced recurrence-free survival. Malignant tumors exhibit earlier and more frequent recurrences, often with regional spread. These findings emphasize the importance of adequate preoperative assessment and achievement of clear surgical margins through appropriate radical management in high-risk cases. A risk-adapted, individualized treatment approach is essential for optimizing oncological outcomes while ensuring effective long-term disease control.
